# The S100B-RAGE pathway is dysregulated in the ALS-linked neuroinflammatory process

**DOI:** 10.1186/2193-1801-4-S1-P27

**Published:** 2015-06-12

**Authors:** Fabrizio Michetti, Claudia Donno, Nadia D’Ambrosi

**Affiliations:** Institute of Anatomy and Cell Biology, Università Cattolica S. Cuore, Rome, Italy

**Keywords:** S100B, RAGE, ALS

The comprehension of the mechanisms at the basis of astrocytic dysfunction in ALS is crucial to limit neuronal injury. Most of the toxic astrocytic effects highlight the role of intracellular calcium. S100B is a Ca2+-binding protein particularly present in astrocytes, behaving as a neuroinflammatory mediator as it is secreted by astrocytes under pathological conditions and can display paracrine toxicity by binding to RAGE. During ALS progression S100B increases in patient astrocytes and, in a rat model of the disease, S100B is augmented in “aberrant astrocytes”, characterized by their neurotoxic potential. The induction of S100B in astrocytes, its release and its interaction with RAGE in motoneurons could represent a hazardous mechanism that takes place during ALS. Main objectives of this work were to investigate 1) if the expression of S100B protein and RAGE change during the course of the disease in rodent models of ALS, 2) if the expression of mutant SOD1 protein per se is sufficient to modify S100B levels in astrocytic cultures. We observed that S100B levels and localization are modulated in the spinal cord and in the brain cortex of rat and mouse models of ALS. We also demonstrated a differential expression of RAGE subunits in SOD1-G93A-derived CNS tissues. Moreover, we showed that the overexpression of mutant SOD1 in astrocytic cell line is sufficient to increase the intracellular levels and release of S100B, while it is not enough to induce a differential expression of RAGE. Thus, the expression of mutant SOD1 interferes with the physiological expression of S100B and RAGE and reveals that in astrocytes S100B modulation is an early event related to the mere expression of mutant SOD1, while the dysregulation of RAGE might be a phenomenon possibly requiring a more complex interplay between different cell types and pathways. Overall, these data suggest that S100B might be a toxic mediator released by astrocytes in the ALS-linked neuroinflammatory process.

